# Molecular and serological investigation of 2019-nCoV infected patients: implication of multiple shedding routes

**DOI:** 10.1080/22221751.2020.1729071

**Published:** 2020-02-17

**Authors:** Wei Zhang, Rong-Hui Du, Bei Li, Xiao-Shuang Zheng, Xing-Lou Yang, Ben Hu, Yan-Yi Wang, Geng-Fu Xiao, Bing Yan, Zheng-Li Shi, Peng Zhou

**Affiliations:** a CAS Key Laboratory of Special Pathogens, Wuhan Institute of Virology, Center for Biosafety Mega-Science, Chinese Academy of Sciences, Wuhan, People’s Republic of China; b Wuhan Pulmonary Hospital, Wuhan, People’s Republic of China

**Keywords:** 2019-nCoV, Wuhan pneumonia, epidemiology, swabs, intestine

## Abstract

In December 2019, a novel coronavirus (2019-nCoV) caused an outbreak in Wuhan, China, and soon spread to other parts of the world. It was believed that 2019-nCoV was transmitted through respiratory tract and then induced pneumonia, thus molecular diagnosis based on oral swabs was used for confirmation of this disease. Likewise, patient will be released upon two times of negative detection from oral swabs. However, many coronaviruses can also be transmitted through oral–fecal route by infecting intestines. Whether 2019-nCoV infected patients also carry virus in other organs like intestine need to be tested. We conducted investigation on patients in a local hospital who were infected with this virus. We found the presence of 2019-nCoV in anal swabs and blood as well, and more anal swab positives than oral swab positives in a later stage of infection, suggesting shedding and thereby transmitted through oral–fecal route. We also showed serology test can improve detection positive rate thus should be used in future epidemiology. Our report provides a cautionary warning that 2019-nCoV may be shed through multiple routes.

## Introduction

Coronaviruses (CoVs) belong to the subfamily *Orthocoronavirinae* in the family *Coronaviridae* and the order *Nidovirales*. A human coronavirus (SARS-CoV) caused the severe acute respiratory syndrome coronavirus (SARS) outbreak in 2003. Most recently, an SARS-related CoV was implicated as the etiological agent responsible for the outbreak in Wuhan, central China. This outbreak is estimated to have started on 12th December 2019 and 17,332 laboratory confirmed cases with 361 deaths as of 3rd February 2020 in China [1]. The virus has spread to 23 other countries by travellers from Wuhan [1]. Typical symptoms are fever, malaise, shortness of breath and in severe cases, pneumonia [2–4]. The disease was first called unidentified viral pneumonia.

We quickly identified the etiological agent, termed 2019-nCoV (virus name designated by the World Health Organization). The newly identified virus is an SARS-related virus (SARSr-CoV) but shares only 74.5% genome identity to SARS-CoV [2]. We developed molecular detection tools based on viral spike genes. Our previous studies indicate that qPCR method can be used for the detection of 2019-nCoV in oral swabs or in bronchoalveolar lavage fluid (BALF) [5]. Additionally, we developed IgM and IgG detection methods using a cross-reactive nucleocapsid protein (NP) from another SARSr-CoV Rp3 [6], which is 92% identical to 2019-nCoV NP. Using these serological tools, we demonstrate viral antibody titres increase in patients infected with 2019-nCoV [5].

Like SARS-CoV, 2019-nCoV induced pneumonia through respiratory tract by clinical observation. Therefore, the presence of viral antigen in oral swabs was used as detection standard for 2019-nCoV. Similarly, two times of oral swabs negative in a 24-h interval was considered as viral clearance by patients officially. Here we launched an investigation of 2019-nCoV in a Wuhan hospital, aiming to investigate the other possible transmission route of this virus.

## Materials and methods

### Sample collection

Human samples, including oral swabs, anal swabs and blood samples were collected by Wuhan pulmonary hospital with the consent from all patients and approved by the ethics committee of the designated hospital for emerging infectious diseases. Two investigations were performed. In the first investigation, we collected samples from 39 patients, 7 of which were in severe conditions. In the second investigation, we collected samples from 139 patients, yet their clinical records were not available. We only showed patients who were viral nucleotide detection positive. Patients were sampled without gender or age preference unless where indicated. For swabs, 1.5 ml DMEM+2% FBS medium was added in each tube. Supernatant was collected after 2500 rpm, 60 s vortex and 15–30 min standing. Supernatant from swabs were added to lysis buffer for RNA extraction. Serum was separated by centrifugation at 3000 *g* for 15 min within 24 h of collection, followed by 56°C 30 min inactivation, and then stored at 4°C until use.

### RNA extraction and qRT-PCR

Whenever commercial kits were used, manufacturer’s instructions were followed without modification. RNA was extracted from 200 μl of samples with the High Pure Viral RNA Kit (Roche). RNA was eluted in 50 μl of elution buffer and used as the template for RT–PCR. QPCR detection method based on 2019-nCoV S gene can be found in the previous study [5]. In brief, RNA extracted from above used in qPCR by HiScript® II One Step qRT-PCR SYBR® Green Kit (Vazyme Biotech Co., Ltd). The 20 μl qPCR reaction mix contained 10 μl 2× One Step SYBR Green Mix, 1 μl One Step SYBR Green Enzyme Mix, 0.4 μl 50 × ROX Reference Dye 1, 0.4 μl of each primer (10 μM) and 2 μl template RNA. Amplification was performed as follows: 50°C for 3 min, 95°C for 30 s followed by 40 cycles consisting of 95°C for 10 s, 60°C for 30 s, and a default melting curve step in an ABI 7500 machine.

### Serological test

In-house anti-SARSr-CoV IgG and IgM ELISA kits were developed using SARSr-CoV Rp3 NP as antigen, which shared above 90% amino acid identity to all SARSr-CoVs, as reported previously [5]. For IgG test, MaxiSorp Nunc-immuno 96 well ELISA plates were coated (100 ng/well) overnight with recombinant NP. Human sera were used at 1:20 dilution for 1 h at 37°C. An anti-Human IgG-HRP conjugated monoclonal antibody (Kyab Biotech Co., Ltd, Wuhan, China) was used at a dilution of 1:40,000. The OD value (450–630) was calculated. For IgM test, MaxiSorp Nunc-immuno 96 wellELISA plates were coated (500 ng/well) overnight with anti-human IgM (µ chain). Human sera were used at 1:100 dilution for 40 min at 37°C, followed by anti-Rp3 NP-HRP conjugated (Kyab Biotech Co., Ltd, Wuhan, China) at a dilution of 1:4000. The OD value (450–630) was calculated.

## Results

In the first investigation, we aimed to test whether viral positive can be found in anal swab and blood as well as oral swabs. We conducted a molecular investigation to patients in Wuhan pulmonary hospital, who were detected as oral swabs positive for 2019-nCoV upon admission. We collected blood, oral swabs and anal swabs for 2019-nCoV qPCR test using previously established method [5].

We found 15 patients who still carry virus following days of medical treatments. Of these patients, 8 were oral swabs positive (53.3%), 4 were anal swabs positive (26.7%), 6 blood positives (40%) and 3 serum positives (20%). Two patients were positive by both oral swab and anal swab, yet none of the blood positive was also swabs positive. Not surprisingly, all serum positives were also whole serum positive (Table 1). In summary, viral nucleotide can be found in anal swab or blood even if it cannot be detected in oral swabs. It should be noted that although swabs may be negative, the patient might still be viremic.

**Table 1.** Molecular detection of 2019-nCoV in swabs and blood. Samples were from oral swabs (OS), anal swabs (AS) and blood. Data were shown as qPCR Ct values. Patients in severe condition during investigation were shown.

	OS	AS	Whole blood	Serum	Severe disease
Patient 1	33.5				No
Patient 2			30.3	24.3	Yes
Patient 3	30.3				No
Patient 4			32.1		No
Patient 5		33.1			No
Patient 6			30.6		No
Patient 7	32.7	30.2			No
Patient 8		33.1			No
Patient 9			31.4	34.5	No
Patient 10			30.9	33.0	Yes
Patient 11	27.3				No
Patient 12	34.4				Yes
Patient 13	32.9	33.6			No
Patient 14	32.3				No
Patient 15			31.6		No

**Figure 1.**
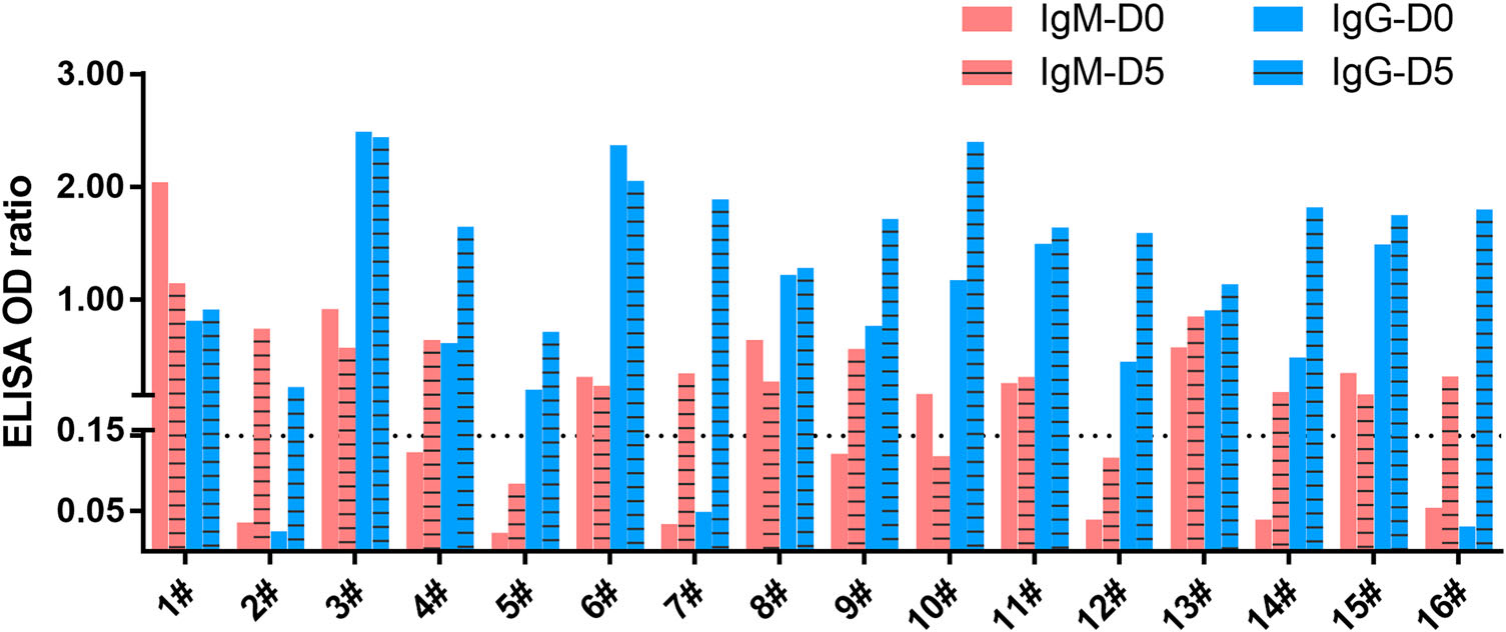
Serological detection of 2019-nCoV. Dashed line indicates cutoff, which was determined based on data from healthy controls.

We then did another investigation to find out the dynamic changes of viral presence in two consecutive studies in both oral and anal swabs in another group of patients. The target patients were those who received around 10 days of medical treatments upon admission. We tested for both viral antibody and viral nucleotide levels by previously established method [5]. We showed that both IgM and IgG titres were relatively low or undetectable in day 0 (the day of first sampling). On day 5, an increase of viral antibodies can be seen in nearly all patients, which was normally considered as a transition from earlier to later period of infection (Figure 1 and supplementary table 1). IgM positive rate increased from 50% (8/16) to 81% (13/16), whereas IgG positive rate increased from 81% (13/16) to 100% (16/16). This is in contrast to a relatively low detection positive rate from molecular test (below).

For molecular detection, we found 8 oral swabs positive (50%) and 4 anal swabs (25%) in these 16 people on day 0. On day 5, we were only able to find 4 oral swabs positive (25%). In contrast, we found 6 anal swabs positive (37.5%). When counting all swab positives together, we found most of the positives came from oral swab (8/10, 80%) on day 0. However, this trend appears to change on day 5. We found more (6/8, 75%) anal swab positive than oral swab positive (4/8, 50%). Another observation is the reoccurrence of virus in 6 patients who were detected negative on day 0. Of note, 4 of these 6 viral positives were from anal swabs (Table 2). These data suggested a shift from more oral positive during early period (as indicated by antibody titres) to more anal positive during later period might happen.

**Table 2.** Molecular detection of 2019-nCoV in swabs from two investigations. Samples were from oral swabs (OS), anal swabs (AS) and blood. Data were shown as qPCR Ct values.

	Date 0-OS	Date 0-AS	Date 5-OS	Date 5-AS
Patient 1			23.2	
Patient 2	30.3			
Patient 3		19.5		
Patient 4	32.7	30.2		
Patient 5		33.1		
Patient 6	31.1		30.0	31.4
Patient 7	27.3			
Patient 8			27.0	
Patient 9	32.9	33.6		
Patient 10				23.8
Patient 11	31.9			
Patient 12	32.3			
Patient 13				17.8
Patient 14				25.5
Patient 15				30.0
Patient 16	33.8		26.9	27.5

## Discussion

Within 1 month of the 2019-nCoV disease outbreak, we rapidly developed molecular and serological detection tools. This is the first molecular and serological study on this virus after the initial identification of 2019-NCoV from 7 patients diagnosed with unidentified viral pneumonia [5]. We detected the virus in oral swabs, anal swabs and blood, thus infected patients can potentially shed this pathogen through respiratory, fecal–oral or body fluid routes. In addition, we successfully applied serology test a large population and showed which could greatly improved detection positive rate.

We show that the current strategy for the detection of viral RNA in oral swabs used for 2019-nCoV diagnosis is not perfect. The virus may be present in anal swabs or blood of patients when oral swabs detection negative. In SARS-CoV and MERS-CoV infected patients, intestinal infection was observed at later stages of infection [7–9]. However, patients infected with 2019-nCoV may harbour the virus in the intestine at the early or late stage of disease. It is also worth to note none of the patients with viremia blood had positive swabs. These patients would likely be considered as 2019-nCoV negative through routine surveillance, and thus pose a threat to other people. In contrast, we found viral antibodies in near all patients, indicating serology should be considered for 2019-nCoV epidemiology. A possible shift from oral positive during early infection to anal swab positive during late infection can be observed. This observation implied that we cannot discharge a patient purely based on oral swabs negative, who may still shed the virus by oral–fecal route. Above all, we strongly suggest using viral IgM and IgG serological test to confirm an infection, considering the unreliable results from oral swabs detection.

In summary, we provide a cautionary warning that 2019-nCoV may be transmitted through multiple routes. Both molecular and serological tests are needed to definitively confirm a virus carrier.

## Supplementary Material

Supplemental Material
